# Multiple non-invasive peripheral vascular function parameters with obesity and cardiometabolic risk indicators in school-aged children

**DOI:** 10.1186/s12887-022-03214-4

**Published:** 2022-03-19

**Authors:** Wennan He, Yi Zhang, Xuesong Li, Kai Mu, Yalan Dou, Ying Ye, Fang Liu, Weili Yan

**Affiliations:** 1 Department of Clinical Epidemiology, Children’s Hospital of Fudan University, National Children’s Medical Center, 399 Wanyuan Road, 201102 Shanghai, P.R. China; 2 Department of General Surgery, Shanghai No. 5 People’s Hospital of Fudan University, Shanghai, P.R. China; 3 Pediatric Heart Center, Children’s Hospital of Fudan University, National Children’s Medical Center, Shanghai, P.R. China

**Keywords:** Endo-PAT, Obesity, BaPWV, Pediatrics, Vascular assessment

## Abstract

**Background:**

The Peripheral Arterial Tonometry (PAT) technique measured by Endo-PAT™, is recently introduced for peripheral vascular assessment in youth, primarily benefits from its easy and non-invasive operation. However, the value of Endo-PAT as early indicator of obesity-related cardiometabolic risk factors remains unclear, with few studies focusing solely on Reactive Hyperemia Index (RHI). A wider coverage of Endo-PAT algorithms is recommended to be applied simultaneously in youth. We evaluated the value of multiple Endo-PAT parameters on obesity and cardiometabolic risk indication in school-aged children, in comparison with another non-invasive Brachial-ankle Pulse Wave Velocity (BaPWV) method.

**Methods:**

This cross-sectional sample included 545 youth (80 with overweight and 73 with obesity) aged 7–17 years. RHI, Framingham-Reactive Hyperemia Index (F-RHI), peak response and Augmentation Index normalized to Heart Rate 75 bpm (AIx75) were measured by Endo-PAT™ 2000 device. Spearman correlations of abovementioned Endo-PAT parameters and BaPWV, with adiposity (weight, waist circumference, BMI, body fat mass) and cardiometabolic indicators (glycemic response, blood pressure, lipid profiles) were calculated with non-linear adjustment on age, height, gender and baseline pulse-wave amplitude (PWA) using fractional polynomials. Analysis was repeated in students with obesity only [median BMI z score: 3.0 (2.5,3.5)] for sensitivity analysis.

**Results:**

The correlations of Endo-PAT parameters with adiposity measures and cardiometabolic indicators were overall mixed and weak (DBP: r ranged from − 0.20 to − 0.13, others: |r| < 0.1) after adjustment. Except that body fat mass (AIx75: *r* = 0.52 *p* < 0.01) and triglyceride level (RHI: *r* = − 0.32 *p* < 0.01, F-RHI: *r* = − 0.21 *p* > 0.05) was moderately reversed in students with obesity. In contrast, BaPWV showed consistently moderate correlations (|r| ranged from 0.123 to 0.322, *p* < 0.05) with almost all adiposity measures and cardiometabolic indicators regardless of obesity status.

**Conclusion:**

Contrary to previous suggestion, various Endo-PAT parameters performed similarly weak for early cardiometabolic risk indication in school-aged children, and less preferable than that by another non-invasive BaPWV method. Despite further investigation is needed to improve certainty of relevant research evidence, innovative technology and algorithms taking into account specifics of young population are worthy of consideration.

**Supplementary Information:**

The online version contains supplementary material available at 10.1186/s12887-022-03214-4.

## Background

Impaired endothelium is reported to edge up as early as 7 years of age [1]. Precocious endothelial dysfunction can be detected in adolescents, and worsen under poor metabolic control [2, 3] highlighting the need for early screening on abnormal arteries. Yet, as a well-proved cardiovascular risk factor contributing to impaired endothelial function and arterial compliance in adults [4–8] the impact of obesity on vascular function during childhood is still in debate, followed by mixed findings with sparse data [9]. Similarly, arterial stiffness may begin during adolescence, as reflected by an increase in brachial-ankle pulse wave velocity (BaPWV) with age, particularly in 15–17 years old [10, 11]. However, obesity-related index with arterial reactivity in these two studies showed conflicting results, as one [11] found strong correlation while the other did not [10] The disagreement arose because of differential assessment tools, the degree of obesity, or methods to normalize data, and more importantly, be masked by pubertal development. Unlike adult obesity, child obesity might cause an earlier peak in vascular compliance, as a result of growth and maturation [9, 12]. Driven by a rapid increase in prevalence of child obesity worldwide, the shift of attention on vascular assessment in pediatric population is worthy to take a precedence in order to control obesity-related metabolic consequences as early as possible.

The scientific experience on vascular assessment in pediatric population lags behind the evidence supported in adults [13] The potential for assessing fingertip microvasculature by Peripheral Arterial Tonometry (PAT) using Endo-PAT™ device in children is recently introduced, primarily benefits from its non-invasive, easy operation, and high reproducibility [4]. Nonetheless, whereas good repeatability and reliability of Endo-PAT among children and adolescents [13, 14] the value of Endo-PAT parameters as indicator of obesity-related cardiometabolic risk factors remains unclear, with few studies focusing solely on automatically calculated reactive hyperemia index (RHI). A wider coverage of Endo-PAT parameters is recommended to be reported in young population [13] However, this was not well explored before.

The current study evaluated the value of multiple Endo-PAT parameters on obesity and cardiometabolic risk indication in children and adolescents, aiming to explore if alternative Endo-PAT parameters performed better indicative value than exclusively RHI use in pediatric population.

## Methods

### Participants

Our subjects were recruited between September 2014 to May 2015 from the Minhang province of Shanghai, which were nested in the 2013–2015 China Child and Adolescent Cardiovascular Health (CCACH) Study [15]. The CCACH study was a nationwide cross-sectional study covering Chinese children and adolescents. The full criteria of participants were described elsewhere [15]. Students aged 7–17 years with complete demographic, anthropometric, blood pressure, biochemical measurement, and free of congenital heart diseases, diabetes mellitus, peripheral vascular diseases or hypertension were invited into this study. Among respondents whose parents agreed to participate, a cluster sampling was conducted by enrolling at least 40 students per age group with balanced sex.

The enrollment was on-going until the target sample size of 540 in total was reached, which ensured power > 0.8 for correlations with r > = 0.12 and alpha of 5%.

### Obesity and cardiometabolic indicators

Data of demographic information (gender, age), anthropometric measurements and cardiometabolic indicators were directly extracted from CCACH database [15]. All measurement were performed at school levels. Height(cm), waist circumference(cm) (WC) and weight (kg) were measured to the nearest 0.5 cm or 0.5 kg with bare foot and light clothing, using standard stadiometers and weight scales in adherence to protocols of the Physical Fitness and Health Surveillance of Chinese School Students [15]. BMI was calculated as body weight (kg) divided by squared height (m^2^), and BMI SD score was further computed [16]. Students with overweight and obesity were identified according to national classification criteria [Table 10 in [17]]. Blood pressure were assessed on the right arm with an appropriately sized cuff using an oscillometric device (HEM-7012, Omron Healthcare, Kyoto, Japan) after at least 5 min’ rest. The measurements were repeated three times in at least 30-s intervals and the mean values were used. Body fat mass and serum insulin levels were available in 182 sub-samples aged 15–17 years. Body fat mass (%) was measured by a trained and qualified nurse using dual energy X-ray absorptiometry (DEXA, Hologic, Inc., Bedford, MA). Serum insulin levels were measured by chemiluminescence methods using Human Insulin kits purchased from Beckman Coulter (California, USA). Fasting glucose and lipid profiles (TC, Total Cholesterol; TG, Triglycerides; HDLC, high density lipoprotein cholesterol; LDLC, low-density lipoprotein cholesterol) were available in 512 subjects. All blood biochemistries were measured on the morning following an overnight fast. Homeostasis model of assessment for insulin resistance index (HOMA-IR) [fasting insulin (μIU/ml) × fasting glucose (mmol/L)/22.5] [18] was also calculated.

### Vascular function

Vascular function was assessed at school levels within 2 weeks after subjects’ enrollment. Endo-PAT™ 2000 device (Itamar Medical Limited, Israel) [19] was applied in adherence to official device operation manual. The detailed description of Endo-PAT application can also be found in a recent methodological review [13]. Briefly, Endo-PAT™ is a computer-based system based on the use of PAT technology, which measures post-ischemic vascular responsiveness following upper arm blood flow occlusion. The device comprises two pneumatic probes for fingertip plethysmograph on both hands that register arterial pulse wave amplitudes (PWAs) at 5-min baseline assessment. In case of concern on bad fit of probe in young children, a quality control took place during the assessment to make sure all subjects’ fingertip could be inserted all the way to the end of the probe. Then the brachial artery of the non-dominant arm is occluded by inflating a sphygmomanometer to supra-systolic pressure for 5 min until cuff release, and another 5-min post-deflation hyperemia is recorded. RHI is automatically calculated as the ratio of average PWAs in 90–150 s post-deflation duration to the baseline average PWAs in the occluded arm compared to the control arm, which reflects pressure changes by the reactive hyperemia. The Framingham reactive hyperemia index (F-RHI) is another automatically outputted PAT index derived from Framingham Heart Studies [13]. It is the natural log transform of the same ratio as RHI, while without a correction of occluded baseline amplitude [0.2276 ∗ ln(mean occluded baseline amplitude) − 0.2], and is using shorter post occlusion times (90–120 s). In addition, we manually calculated the peak response that used the maximum post-deflation PWAs among averages of each 30 s amplitude intervals instead of a fixed post-occlusion duration. Peak response can better reflect the “true” peak hyperemic response of children and adolescents as time-to-peak is delayed in this population compared to that in adults [13]. Higher PAT ratio reflects better endothelial function.

Endo-PAT™ not only measures endothelial function but also assesses arterial stiffness by measuring the peripheral augmentation index (AIx) from the radial pulse wave analysis. AIx indicates the proportion of difference between backward reflected peak (P2) and systolic peak (P1) by P1. Lower AIx reflects better vascular compliance. Because AIx is heart rate related, the result is corrected to a standard of AIx at heart rate of 75BPM (AIx75).

On the same day, BaPWV was measured as another index of arterial stiffness for comparison (automatic waveform analyzer BP-203RPE-I; Colin Medical Technology, Komaki, Japan). Before measurement, four cuffs on both brachia and ankles were fitted with oscillometric sensors [20] with at least 10 min’ rest at supine position in a dedicated, somber and quiet testing room (22 ± 2 °C). The time intervals (T) between the wave fronts of the brachia and those of the ankles were calculated. The distance (L) between the heart and sampling points was calculated automatically according to the subjects’ height. BaPWV is calculated using the following formula: BaPWV = (La-Lb) /T, where ‘La’ is the path length from the heart to the ankle and ‘Lb’ is the path length from the heart to the brachium.

Two trained and experienced operators completed the vascular assessment blinded to other clinical data and did not participant in the following analysis and manuscript preparation.

### Statistical analysis

Characteristics of study population were summarized in all and by obesity status. Student’s t tests and chi-square tests were performed for group comparisons in continuous and categorical variables. We summarized age-dependent trends of Endo-PAT parameters (RHI, F-RHI, Peak response, AIx75) in all and by BMI groups, using two-way fractional polynomial prediction plots. Because of few students with obesity especially in girls, groups with overweight and obesity were combined in order to generate a smoother function of growth with age.

We performed two correlation analysis of Endo-PAT parameters with age, adiposity measures (weight, height, BMI, WC, and body fat mass) and cardiometabolic indicators (blood pressure, glycemic and lipid profiles). First was bivariate correlation by spearman method. Second was generated independent of baseline somatic growth, by using fractional polynomial regression analysis.

Fractional polynomials have been validated to predict non-linear growth trajectories [21]. Each Endo-PAT parameter was modelled by function of age, where height, gender and baseline PWAs of occluded arm were covariates. The model deviance of powers was chosen from the set {− 2, − 1, − 0.5, 0, 0.5, 1, 2, 3} (a power of zero is the log function) to identify the best-fitted fractional polynomial. Fractional polynomial was generated by “fp” function in STATA 15.1 SE (Stata, College Station, TX). By default, “fp” will fit degree-2 factional polynomial (FP2) model and compared it with degree-1(FP1) model by χ _2_ distribution with 2 degrees of freedom. If FP2 significantly improves the model and higher-degree models do not further alter the model, we accept FP2 model otherwise we instead it of FP1 model. Using RHI as an example, FP1 and FP2 model could be formulated as [21]:

1 $$\mathrm{FP}1: RHI={\beta}_0+{\alpha}_0 Covariate+\left({\beta}_1+{\alpha}_1 Covariate+{\mu}_1\right){Age}^{p1}+{\mu}_0+\varepsilon$$

2 $$\mathrm{FP}2:\kern0.5em \mathrm{If}\ \mathrm{p}1\ne \mathrm{p}2: RHI={\beta}_0+{\alpha}_0 Covariate+\left({\beta}_1+{\alpha}_1 Covariate+{\mu}_1\right){Age}^{p1}+\left({\beta}_2+{\alpha}_2 Covariate+{\mu}_2\right){Age}^{p2}+{\mu}_0+\varepsilon$$

3 $$\mathrm{If}\ \mathrm{p}1=\mathrm{p}2: RHI={\beta}_0+{\alpha}_0 Covariate+\left({\beta}_1+{\alpha}_1 Covariate+{\mu}_1\right){Age}^{p1}+\left({\beta}_2+{\alpha}_2 Covariate+{\mu}_2\right){Age}^{p2}\log (Age)+{\mu}_0+\varepsilon$$

p: power.

β: fixed coefficients describing the average shape of the trajectory.

μ: deviation of trajectory from average.

After fitting the power(s) for each model, individual-specific occasion level residuals, representing the deviation from fitted trajectory, were used for the second correlation analysis. The results were therefore modelled against baseline somatic growth, of which we decided were age, gender, height and baseline PWA. Of note is that, the abovementioned correlation analysis was also performed in BaPWV, in comparison with results of Endo-PAT parameters. As vascular response with growth and metabolic profiles might perform differently in children and adolescents with severely high BMI [10, 22], we repeated the above correlation analysis limited in students with obesity.

All analyses were carried out using STATA 15.1 SE (Stata, College Station, TX). A significant level was set at two-sided *p*-value of < 0.05. Multiple testing correction was not performed in these analyses.

## Results

### Characteristics of participants

Characteristics of 545 subjects were presented in all and by groups of non-OB (non-overweight or -obese, *n* = 392), overweight (*n* = 80) and obesity (*n* = 73) in Table 1. Mean age was 12(±3) with a range of 7–17 years. 296(54.3%) were males and the proportion increased in group of overweight (58.8%) and obesity (78.1%). Thirty-seven of seventy-three subjects with obesity had BMI SD score ≥ 3. Students with overweight and obesity had higher WC, body fat mass, blood pressure levels, glycemic levels (fasting glucose, insulin levels, HOMA-IR), TC, TG, LDL-C and lower HDL-C than non-OB group. BaPWV(*p* = 0.001) and baseline PWA(*p* < 0.001) were significantly higher in students with overweight and obesity. Despite lack of significance, F-RHI, peak response and Aix75 were lower, and RHI was higher in individuals with overweight and obesity.

**Table 1:** Characteristics of study subjects^a^

	Overall *N* = 545	Non-OB *N* = 392	Overweight *N* = 80	Obese *N* = 73	***P*** value
**Demographic characteristics**					
Male, n (%)	296(54.3%)	192(49.0%)	47(58.8%)	57(78.1%)	< 0.001
Age (years)	12 (3)	12 (3)	12 (3)	12 (3)	0.742
Weight (kg)	46.7(18.4)	41.2(13.5)	54.3(16.8)	68.0(24.1)	< 0.001
Height (cm)	151.0(17.0)	149.9(17.0)	153.1(16.8)	155.1(16.7)	0.027
BMI (kg/m^2^)	19.7(4.5)	17.7(2.7)	22.4(2.6)	27.2(4.7)	< 0.001
BMI SD score^d^	0.2(−0.5,1.3)	−0.2(−0.7,0.3)	1.6(1.3,1.9)	3.0(2.5,3.5)	< 0.001
WC, cm	67.5(12.6)	62.2(7.8)	74.3(8.1)	88.1(12.1)	< 0.001
Body fat percentage %^b^	28.9(7.5)	26.9(7.2)	32.0(6.6)	36.0(3.9)	< 0.001
**Cardiovascular characteristics**					
SBP (mmHg)	116(13)	113(11)	123(13)	129(12)	< 0.001
DBP (mmHg)	67(9)	65(8)	70(8)	73(8)	< 0.001
Heart Rate	79(11)	79(11)	79(11)	82(12)	0.108
Baseline PWA (occ) (au)^d^	169(87,331)	148(77,277)	161(92,346)	285(1741,481)	< 0.001
Baseline PWA (con) (au)^d^	171(91,340)	144(84,296)	185(97,352)	317(170,504)	< 0.001
RHI (ml/mm Hgx100)	1.66(0.58)	1.64(0.53)	1.69(0.68)	1.72(0.71)	0.507
InRHI(ml/mm Hgx100^−1^)	0.45(0.33)	0.45(0.32)	0.45(0.37)	0.48(0.36)	0.791
F-RHI(ml/mm Hgx100^−1^)	0.52(0.39)	0.55(0.38)	0.49(0.44)	0.44(0.41)	0.064
Peak response^d^ (ml/mm Hgx100)	1.84(1.29,2.57)	1.86(1.31,2.61)	1.76(1.20,2.41)	1.68(1.28,2.34)	0.308
AIx75 (%)	−8.49(− 10.71)	− 8.02(10.43)	− 10.23(11.79)	− 9.12(10.84)	0.215
BaPWV(mm/s)	861(127)	849(116)	883(154)	903(143)	0.001
**Glycemic and lipid profiles**					
Fasting Glucose (mmol/L)^c^	5.1(0.6)	5.1(0.6)	5.1(0.8)	5.3(0.7)	0.047
Insulin (μIU/ml)^b, d^	5.5(3.8,7.9)	4.8(3.6,6.7)	6.4(3.9,9.2)	11.9(8.0,26.9)	< 0.001
HOMA-IR^b, d, e^	1.3(0.8,1.8)	1.1(0.8,1.5)	1.5(1.0,2.1)	2.6(1.8,6.1)	< 0.001
TC (mmol/L)^c^	3.99(0.70)	3.94(0.66)	3.97(0.72)	4.30(0.85)	< 0.001
TG (mmol/L)^c^	0.67(0.37)	0.59(0.29)	0.75(0.33)	1.09(0.52)	< 0.001
HDLC (mmol/L)^c^	1.45(0.29)	1.51(0.28)	1.33(0.26)	1.30(0.23)	< 0.001
LDLC (mmol/L)^c^	2.36(0.63)	2.27(0.57)	2.47(0.62)	2.73(0.76)	< 0.001

*Abbreviations*: *Non-OB* subjects without overweight or obese status, *BMI* Body mass index, *WC* Waist circumference, *SBP* Systolic blood pressure, *DBP* diastolic blood pressure, *PWA* pulse-wave amplitude, *occ* occlusion arm, *con* control arm, *RHI* reactive hyperemia index, *F-RHI* Framingham-reactive hyperemia index, *AIx75* Augmentation Index normalized to Heart Rate 75 bpm, *BaPWV* Brachial-ankle pulse wave velocity, *TC* Total Cholesterol, *TG* Triglycerides, *HDLC* high density lipoprotein cholesterol, *LDLC* low-density lipoprotein cholesterol

^a^summarized as mean (standard deviation) if not specified

^b^available in 182 subjects

^c^available in 512 subjects

^d^median (25th,75th)

^e^HOMA-IR = [fasting insulin (μIU/ml) × fasting serum glucose (mmol/L)]/22.5

### Age-dependent trends of Endo-PAT parameters

**Fig. 1:**
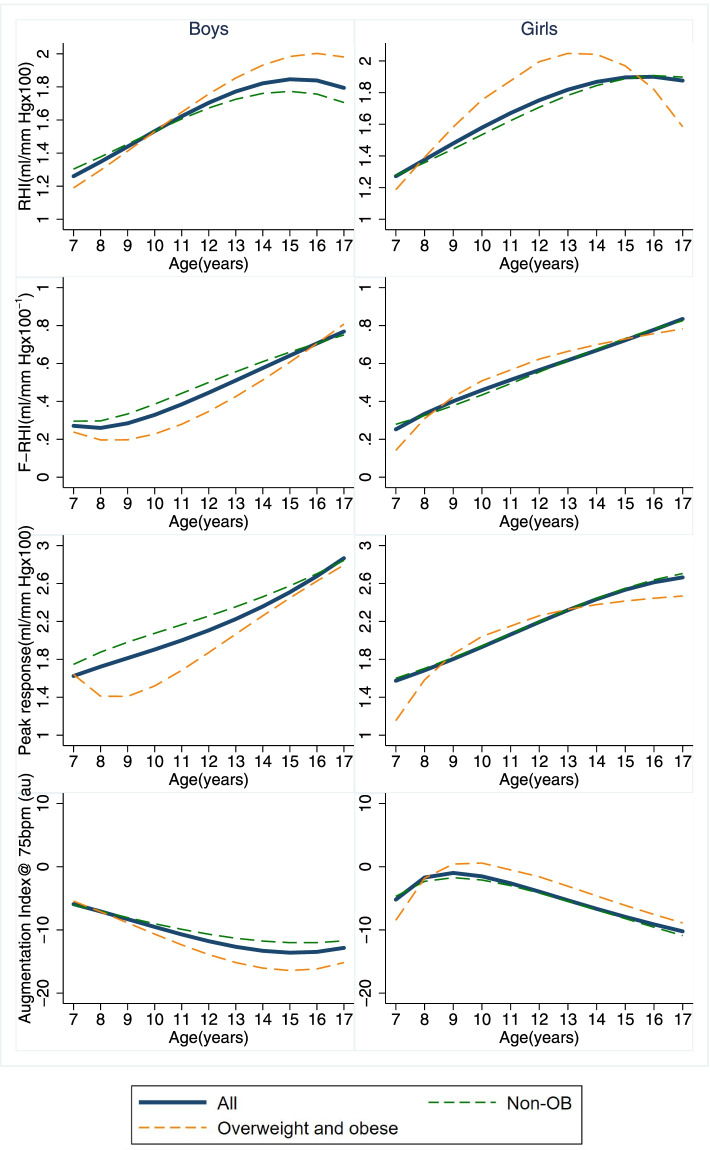
Fractional polynomial predicted age-dependent trends of endo-PAT parameters

Figure 1 depicts the age-dependent trends of Endo-PAT parameters. Overall, the trends showed that RHI, F-RHI and peak response were higher and AIx75 were lower in elder students in both genders. The trends appeared to slow down during adolescence, especially in students over 15 years, compared to lower-grade children. When stratified by groups, students with overweight and obesity showed similar trends as non-OB students.

### Bivariate correlation analysis

All parameters were significantly correlated with age, weight, height, WC and BMI in all (all *p* < 0.001). The correlations were also shown as significant when limited in subjects with obesity, except for height with BaPWV (Table 2). RHI, F-RHI, peak response and BaPWV were positively correlated, while AIx75 showed negative correlation with the abovementioned indicators with moderate magnitude. Stronger correlations were observed in students with obesity. RHI, F-RHI and peak response were not correlated, while AIx75 (*r* = 0.154 and 0.573 in all and obese only) and BaPWV (*r* = 0.109 and 0.408 in all and obese only) showed significant correlation with body fat mass (*p* < 0.05), with stronger correlations in students with obesity.

**Table 2:** Bivariate Correlations of endo-PAT parameters, BaPWV with adiposity and cardiometabolic indicators

	Correlation coefficient									
	RHI		F-RHI		Peak response		AIx75		BaPWV	
	All	Obese	All	Obese	All	Obese	All	Obese	All	Obese
Adiposity measures										
age	0.364***	0.394***	0.483***	0.471***	0.352***	0.311**	−0.249***	−0.260*	0.321***	0.376**
weight	0.349***	0.426***	0.345***	0.441***	0.262***	0.280*	− 0.302***	− 0.434***	0.261***	0.263*
height	0.343***	0.407**	0.388***	0.422***	0.282***	0.280*	−0.322***	− 0.402***	0.210***	0.188
WC	0.269***	0.471***	0.192***	0.461***	0.131**	0.295*	−0.229***	− 0.395***	0.254***	0.238*
BMI	0.251***	0.457***	0.188***	0.460***	0.152***	0.302**	−0.209***	−0.410***	0.237***	0.336**
Body fat^a^	0.138	0.070	0.099	0.297	0.039	0.200	0.154*	0.573**	0.109	0.408*
Blood pressure										
SBP	0.053	0.094	0.025	0.043	−0.017	0.057	−0.112**	−0.265*	0.311***	0.322**
DBP	−0.089*	−0.216	− 0.113**	−0.194	− 0.122**	−0.230*	− 0.049	0.141	0.316***	0.198
Glycemic response										
Glu	0.104*	0.267*	0.035	0.151	−0.0003	0.107	− 0.102*	0.080	0.0032	−0.086
Ins^a^	0.039	−0.159	−0.085	− 0.116	−0.003	0.018	0.087	−0.051	0.195**	0.293
HOMA-IR^a^	0.029	−0.114	−0.111	− 0.103	−0.026	0.013	0.076	−0.092	0.191**	0.296
Lipid profile										
TC^b^	−0.038	− 0.146	− 0.066	−0.151	− 0.099*	−0.137	0.151***	0.179	0.044	0.085
TG^b^	0.150***	−0.225	0.105*	− 0.193	0.089*	−0.155	−0.021	0.047	0.168***	0.149
HDLC^b^	0.070	0.208	0.117**	0.202	0.066	0.042	0.126**	0.047	−0.061	0.038
LDLC^b^	0.021	−0.088	−0.027	− 0.114	−0.070	− 0.088	0.059	0.174	0.085	0.064

*Abbreviations*: *RHI* reactive hyperemia index, *F-RHI* Framingham-reactive hyperemia index, *AIx75* Augmentation Index normalized to Heart Rate 75 bpm, *BaPWV* Brachial-ankle pulse wave velocity, *BMI* body mass index, *WC* waist circumference, *SBP* systolic blood pressure, *DBP* diastolic blood pressure, *Glu* fasting glucose, *Ins* insulin level, *TC* Total Cholesterol, *TG* Triglycerides, *HDLC* high density lipoprotein cholesterol, *LDLC* low-density lipoprotein cholesterol

^a^data were available in 182 subjects

^b^data were available in 512 subjects

^*^p < 0.05

^**^p < 0.01

^***^p < 0.001

The correlations with blood pressure, glycemic and lipid profiles were relatively weak and mixed in all parameters (Table 2). RHI, F-RHI and peak response were not correlated with SBP, but showed inverse correlation with DBP (*p* < 0.05). In contrast, AIx75 and BaPWV were significantly correlated with SBP (*p* < 0.05), and BaPWV was also correlated with DBP (*r* = 0.316 *p* < 0.001 in all and *r* = 0.198 in obese only). Notably, all parameters except AIx75 were significantly positively correlated with TG (RHI: *r* = 0.150; F-RHI: *r* = 0.105; peak response: *r* = 0.089; BaPWV: *r* = 0.168), and the directions of correlations in RHI, F-RHI, peak response were reversed in students with obesity (RHI: *r* = − 0.225; F-RHI: *r* = − 0.193; peak response: *r* = − 0.155).

### Correlation analysis independent of baseline somatic growth

As shown in Table 3, the best-fitted curved powers for RHI, F-RHI, peak response, AIx75 and BaPWV was − 0.5, − 0.5, − 0.5, − 2 and {0,0.5} correspondingly. An overall 14, 13 and 19% of variance in RHI, F-RHI and BaPWV could be explained by age, height, gender and baseline PWA. Twenty-four and thirty-nine percent of variance was contributed by the same covariates for peak response and F-RHI respectively. Age was the strongest determinant of RHI (*p* = 0.001), F-RHI (*p* < 0.001), peak response (*p* = 0.010) and BaPWV (*p* < 0.001). Gender (*p* < 0.001) and baseline PWA (*p* = 0.008) were also significant predictors for BaPWV. AIx75 was primarily explained by height and gender (*p* < 0.001).

**Table 3:** Fitted trajectories of endo-PAT and BaPWV with baseline somatic growth, by fractional polynomial regression analysis

Variables	Powers	Coef.	SE.	t	P > |t|	R^2^	Prob > F
**RHI**						0.14	< 0.001
Age	−0.5	−1.310	0.378	−3.47	0.001		
Height		0.002	0.003	0.67	0.504		
Gender		0.076	0.050	1.53	0.127		
Baseline PWA (occ)		0.0002	0.0001	1.28	0.202		
**F-RHI**						0.39	< 0.001
Age	−0.5	− 0.948	0.216	−4.39	< 0.001		
Height		0.003	0.002	1.62	0.107		
Gender		0.040	0.029	1.38	0.167		
Baseline PWA (occ)		−0.0008	0.0001	−12.09	< 0.001		
**Peak response**						0.24	< 0.001
Age	− 0.5	−0.870	0.336	−2.59	0.010		
Height		0.003	0.002	1.14	0.254		
Gender		0.007	0.044	0.16	0.873		
Baseline PWA (occ)		−0.001	0.0001	−9.71	< 0.001		
**AIx75**						0.13	< 0.001
Age	−2	−1.568	1.698	−0.92	0.356		
Height		−0.212	0.549	−3.86	< 0.001		
Gender		4.317	0.922	4.68	< 0.001		
Baseline PWA (occ)		0.0009	0.002	0.40	0.688		
**BaPWV**						0.19	< 0.001
Age	0	− 2104.2	324.3	−6.49	< 0.001		
Age_2	0.5	4266.6	601.4	7.09	< 0.001		
Height		−0.746	0.676	−1.10	0.270		
Gender		−30.92	10.66	−2.90	0.001		
Baseline PWA (occ)		−0.682	0.025	−2.66	0.008		

*Abbreviations*: *PWA* pulse-wave amplitude, *occ* occlusion arm, *RHI* reactive hyperemia index, *FRHI* Framingham-reactive hyperemia index, *AIx75* Augmentation Index normalized to Heart Rate 75 bpm, *BaPWV* Brachial-ankle pulse wave velocity

After accounting for age, height, gender and baseline PWA, the correlations with adiposity measures dropped greatly in all parameters (Table 4). RHI, F-RHI and peak response were only significantly correlated with DBP (RHI: *r* = − 0.132, *p* = 0.002; F-RHI: *r* = − 0.204, *p* < 0.001, peak response: *r* = − 0.176 *p* < 0.001). AIx75 was not correlated with any indicators in all, but with body fat mass in students with obesity (*r* = 0.523, *p* = 0.007). None of Endo-PAT parameters was correlated with glycemic and lipid profiles, except an inverse correlation observed between RHI and TG in students with obesity (*r* = − 0.322, *p* = 0.008). Conversely, although the magnitude decreased, BaPWV was significantly correlated with all adiposity measures and most cardiometabolic indicators (insulin, HOMA-IR, TG, HDL-C, LDL-C), especially for body fat mass, blood pressure and insulin levels. The correlations with cardiometabolic indicators became stronger after adjusting for baseline somatic growth, and consistent in students with obesity.

**Table 4:** Correlations of endo-PAT parameters, BaPWV with adiposity and cardiometabolic indicators independent of baseline growth factors

	Correlation coefficient									
	RHI		F-RHI		Peak response		AIx75		BaPWV	
	All	Obese	All	Obese	All	Obese	All	Obese	All	Obese
Adiposity measures										
weight	−0.004	0.080	0.020	0.096	0.029	0.024	−0.044	−0.162	0.086*	0.042
WC	0.032	0.144	0.026	0.155	0.032	0.077	−0.022	−0.142	0.174***	0.036
BMI	0.027	0.136	0.033	0.127	0.059	0.066	−0.042	−0.176	0.148***	0.130
Body fat^a^	0.103	0.100	0.115	0.116	0.075	0.079	−0.024	0.523**	0.163*	0.474*
Blood pressure										
SBP	−0.036	0.015	−0.055	0.018	−0.066	0.012	0.032	−0.128	0.272***	0.286*
DBP	−0.132**	−0.254*	− 0.204***	−0.121	− 0.176***	−0.169	− 0.021	0.182	0.322***	0.260*
Glycemic response										
Glu	0.027	0.126	0.024	0.047	−0.022	−0.054	−0.024	0.168	0.013	−0.121
Ins^a^	0.011	−0.149	−0.017	− 0.192	0.098	0.095	0.062	−0.047	0.269***	0.409*
HOMA-IR^a^	0.003	−0.104	−0.037	− 0.177	0.076	0.077	0.065	−0.050	0.262***	0.384
Lipid profile										
TC^b^	0.030	−0.064	0.014	−0.115	− 0.029	−0.034	0.067	0.095	0.084	0.179
TG^b^	0.012	−0.322**	0.021	−0.215	0.042	−0.099	0.058	0.204	0.123**	0.136
HDLC^b^	0.031	0.181	0.032	0.097	−0.015	0.013	0.060	0.070	−0.126**	0.070
LDLC^b^	0.045	−0.031	0.032	−0.071	−0.015	0.004	0.016	0.064	0.133**	0.142

*Abbreviations*: *RHI* reactive hyperemia index, *F-RHI* Framingham-reactive hyperemia index, *AIx75* Augmentation Index normalized to Heart Rate 75 bpm, *BaPWV* Brachial-ankle pulse wave velocity, *WC* waist circumference, *SBP* systolic blood pressure, *DBP* diastolic blood pressure, *Glu* fasting glucose, *Ins* insulin level, *TC* Total Cholesterol, *TG* Triglycerides, *HDLC* high density lipoprotein cholesterol, *LDLC* low-density lipoprotein cholesterol

^a^data were available in 182 subjects

^b^data were available in 512 subjects

^*^p < 0.05

^**^p < 0.01

^***^p < 0.001

## Discussion

In this cross-sectional sample of 545 healthy juveniles, we found RHI, F-RHI and peak response went upwards gradually as age increased, and AIx75 went downwards regardless of obesity. All the parameters were significantly correlated with adiposity measures, but the correlations almost disappeared after accounting for age, gender, height and baseline PWA. Their correlations with cardiometabolic indicators were weak and mixed. In contrast, BaPWV showed consistently moderate correlations with nearly all the adiposity measures and cardiometabolic indicators.

Contrary to adults, in which endothelial dysfunction accelerated during ageing, children in the advanced stages of pubertal development had a higher peripheral vasodilatory response compared to pre-pubertal children, as reflected by an increment of PAT index [23, 24]. Our findings were supportive to the physiological advancement of endothelium that RHI increased with age during childhood and adolescence [9, 23, 25, 26]. RHI values were positively correlated with almost all adiposity measures, which was also in line with several studies [27–29]. Childhood obesity appears to stimulate better vascular compliance in growth [9, 12], as an underlying explanation for stronger correlations observed in students with obesity in this study. The development of microvascular function is stimulated by early maturation and not complete until late adolescence [25]. Agarwal et al. found RHI decreased with age in obese adolescents older than 15 years, maintaining this trend in adulthood [30]. In our study, the RHI reached a plateau and started to flop after 15 years old. However, the fitting curves might contain inaccuracy that called for further solid evidence.

In the latest narrative review evaluating Endo-PAT application in paediatric endocrinopathies [31], the authors concluded a low RHI in children and adolescents is more likely to reflect immature juvenile microvascular function rather than dysfunction. The reliability of RHI on predicting adverse cardiometabolic events was limited, given a null association with lipid profiles and insulin resistance in majority of studies. Taken together with our results, the finding regarding RHI was still in the mainstream. Exceptionally, we observed a consistently negative correlation with TG and DBP, especially in students with obesity. Mounting evidence supports that vascular maturation may not be confined to individuals suffering from severe obesity, hypertension, hyperlipidaemia or diabetes mellitus [11]. The value of PAT index in individuals with extremely high BMI or obesity-related complications is worthy of further elaboration. Meanwhile, the various methods to control for puberty status and calculate PAT indexes may tell different stories in regards to vascular function in paediatrics [13, 23, 25, 32, 33]. In short, the value of RHI was still masked by juvenile pubertal development.

The automated algorithms of RHI proposed by Endo-PAT manufacturer have raised confusion across studies because of mixed use of terminologies and post-occlusion time intervals. Driven by this, a wider coverage of PAT indexes is recommended to be simultaneously demonstrated in the same paediatric population [13]. Traditionally, the RHI algorithm was calculated using the PWAs in 90–150 s cuff-deflation which missed the peak responses in 61% of our study subjects, especially adolescents (Additional file 1: Table S1). Yet, instead of RHI, only one small study had used peak response in children and adolescents [22]. In this study, we firstly introduced peak response and F-RHI into analysis in the same paediatric population to see if alternate PAT indexes perform better. Unfortunately, both indexes yielded similar results. F-RHI was reported to be highly associated with cardiovascular risk factors and have better reproducibility in adolescents than RHI [13, 14, 34]. Yet, F-RHI also suffered from missing true peak response because of shorter time interval (90–120 s) and its limited use was exposed in this study. Peak response, though recommended as more reliable than RHI in paediatric population [35], our findings were similar to a previous single study that no significant correlation was found with biochemical cardiovascular indicators [22]. As the underlying biology of reactive hyperemia is partially explained by nitric oxide activity, other unknown risk factors might exist which are not captured by classic cardiometabolic indicators [36, 37]. On balance, our results did not support better validity of F-RHI and peak response in juveniles compared to RHI.

Similarly, results of AIx75 by Endo-PAT technique showed an improvement on arterial elasticity (i.e. AIx75 value decreased) with age and adiposity, while no consistent correlation was identified in terms of lipid and glucose profiles. This was not surprising since a previous study also observed null findings of AIx with any of glucose or lipid profiles in adolescents [38]. Except for body fat mass in students with obesity, where a significantly strong correlation was found. Different measures of adiposity may matter in regards to a link with AIx, as some found arterial stiffness measured by AIx was not preferably predicted by simply BMI, but by body fat content [39] or obesity-related insulin resistance [40, 41]. In comparison, arterial stiffness measured by PWV showed consistently correlations with multiple adverse cardiovascular indicators previously [11, 40, 42] and further confirmed in this study. Especially insulin resistance, which has been reported as a significant influencing factor of PWV and its presence might play a mediating role in relation to obesity [11, 40]. The degeneration of arterial wall was already confirmed in children by BaPWV, yet no correlation with adiposity index was identified in the same population excluding children with severe obesity [10]. In our study, students with obesity (no upper limit was set) showed consistent and even stronger correlation after adjusting for baseline somatic growth. The attempt to improve the current knowledge of vascular pathological change in children with severe obesity is worthy of future exploration, raising the possibility that BaPWV was less influenced by puberty advancement than Endo-PAT parameters. However, as the overall correlations were not strong, the value of BaPWV on early cardiometabolic risk indication was relatively conservative.

### Strength and limitations

To our knowledge, we are the first to build parallel comparisons of alternate PAT algorithms, as well as characteristics of endothelial function and arterial stiffness by Endo-PAT technique in the same young population. This has not been well explored before, and we controlled for somatic growth using effective modelling. Fractional polynomials help detect non-linear trajectories in growth, while the coefficients are not as interpretable as linear models [21]. This study helps narrow the research gap by demonstrating a bigger picture of Endo-PAT value for identifying cardiometabolic risk factors in very early of life. However, we acknowledged that some limitations existed that might affect the results. First was insufficient size by age and gender (Additional file 1: Table S2). Although we reached overall power and pre-specified size for each age, the response rate in certain age was extremely low due to academic pressure during graduation. Besides, few biochemical data were not available in all subjects. These might contribute to the inaccuracy of findings due to limited power calculation in some analysis. However, we believe this might not easily undermine our main conclusion since the whole picture showed overall consistent tendency across different parameters. Secondly, as sex steroids made different contribution on vascular reactivity and endothelial function during puberty [11, 23], puberty stage assessment is preferred to increase validity which was not covered in this study. Our study was cross-sectionally sampled in a certain area. Thus, we highlighted the value of longitudinal study in future investigation, as it exactly deciphers the timing of maturation and pathophysiological characteristics within an individual. Finally, it is noteworthy that there is no established norm of Endo-PAT use in paediatric studies so far [13, 14, 25, 26]. RHI of < 1.67 or 1.35 for adults might mistakenly classify a large proportion of children as dysfunction [25, 43] and analysis in relation to thresholds of Endo-PAT parameters was not considered in this study. Rather, our results raised the possibility that the Endo-PAT system itself was still beyond to be widely applied in general young population, and this could not be easily resolved by alternate PAT algorithms or data normalization.

## Conclusions

Characteristics of Endo-PAT parameters showed better vascular reactivity in children and adolescents as age increased. Multiple Endo-PAT parameters performed similarly weak in obesity-cardiometabolic risk indication, and seemed less preferable than that by another non-invasive BaPWV method. Childhood growth seemed be the largest barrier accounting for limited Endo-PAT application in paediatric studies. Despite further investigation is needed to improve certainty of relevant research evidence, innovative technology and algorithms taking into account specifics of young population are worthy of consideration.

## Supplementary Information

**Additional file 1: Table S1.** Post-occlusion time to peak amplitude. **Table S2.** Number of participants by age, gender and obesity.

## Data Availability

The datasets used and analysed during the current study are available from the corresponding author on reasonable request.

## Abbreviations

Non-OB: Subjects without overweight or obese status
BMI: Body mass index
WC: Waist circumference
SBP: Systolic blood pressure
DBP: Diastolic blood pressure
PWA: Pulse-wave amplitude
occ: Occlusion arm
con: Control arm
RHI: Reactive hyperemia index
F-RHI: Framingham-reactive hyperemia index
AIx75: Augmentation Index normalized to Heart Rate 75 bpm
BaPWV: Brachial-ankle pulse wave velocity
TC: Total Cholesterol
TG: Triglycerides
HDLC: High density lipoprotein cholesterol
LDLC: Low-density lipoprotein cholesterol
