# Supplementary material for: Multiple non-invasive peripheral vascular function parameters with obesity and cardiometabolic risk indicators in school-aged children
Source: BMC Pediatr. 2022 Mar 19;22:146. doi: 10.1186/s12887-022-03214-4 (PMC8934007; doi:10.1186/s12887-022-03214-4)
Supplement: Supplementary file 1 — Additional file 1: Table S1. Post-occlusion time to peak amplitude. Table S2. Number of participants by age, gender and obesity. [file 12887_2022_3214_MOESM1_ESM.docx]

Additional File 1

| **Table S1. Post-occlusion time to peak amplitude** | | | | |
| --- | --- | --- | --- | --- |
| **Time to peak(min)** | **n** | **Age(sd)** | **Male (%)** | **Non-OB (%)** |
| 0-0.5 | 3 | 15(2) | 3(100%) | 1(33.3%) |
| 0.5-1 | 41 | 13(3) | 23(56.1%) | 26(63.4%) |
| 1-1.5 | 106 | 14(2) | 63(59.4%) | 76(71.7%) |
| 1.5-2 | 79 | 13(3) | 40(50.6%) | 61(77.2%) |
| 2-2.5 | 71 | 13(3) | 31(43.7%) | 58(81.7%) |
| 2.5-3 | 63 | 11(3) | 36(57.1%) | 48(76.2%) |
| 3-3.5 | 45 | 11(3) | 21(46.7%) | 28(62.2%) |
| 3.5-4 | 59 | 10(3) | 31(52.5%) | 40(67.8%) |
| 4-4.5 | 50 | 10(3) | 29(58.0%) | 36(72.0%) |
| 4.5-5 | 18 | 11(3) | 16(88.9%) | 9(50.0%) |
| 5-5.5 | 0 | - | - | - |
| 5.5-6 | 0 | - | - | - |
| 6-6.5 | 0 | - | - | - |
| 6.5-7 | 0 | - | - | - |

Abbreviations:

sd, standard deviation; non-OB, subjects without overweight or obese status

| **Table S2. Number of participants by age, gender and obesity** | | | |
| --- | --- | --- | --- |
|  | **Boys (n)/Girls (n)** | | |
| **Age (N=545)** | **Non-OB (N=392)** | **Overweight**  **(N=80)** | **Obese**  **(N=73)** |
| 7 (N=53) | 14/30 | 1/2 | 5/1 |
| 8(N=55) | 22/12 | 9/5 | 5/2 |
| 9(N=61) | 16/20 | 7/7 | 6/5 |
| 10(N=46) | 22/14 | 2/1 | 6/1 |
| 11(N=8) | 4/2 | 1/0 | 1/0 |
| 12(N=31) | 10/10 | 4/2 | 5/0 |
| 13(N=54) | 20/22 | 2/3 | 7/0 |
| 14(N=50) | 19/21 | 5/1 | 2/2 |
| 15(N=70) | 25/24 | 6/5 | 7/3 |
| 16(N=70) | 20/25 | 7/6 | 10/2 |
| 17(N=47) | 20/20 | 3/1 | 3/0 |

Abbreviations:

non-OB, subjects without overweight or obese status.
